# Prospective, randomized trial comparing fluids and dobutamine optimization of oxygen delivery in high-risk surgical patients [ISRCTN42445141]

**DOI:** 10.1186/cc4913

**Published:** 2006-05-12

**Authors:** Suzana M Lobo, Francisco R Lobo, Carlos A Polachini, Daniela S Patini, Adriana E Yamamoto, Neymar E de Oliveira, Patricia Serrano, Helder S Sanches, Marco A Spegiorin, Marcio M Queiroz, Antonio C Christiano, Elisangela F Savieiro, Paula A Alvarez, Silvia P Teixeira, Geni S Cunrath

**Affiliations:** 1 Division of Critical Care Medicine, Departments of Internal Medicine, Anesthesiology and Surgery, Medical School – FUNFARME and Hospital de Base, São José do Rio Preto, São Paulo, Brazil

## Abstract

**Introduction:**

Preventing perioperative tissue oxygen debt contributes to a better postoperative recovery. Whether the beneficial effects of fluids and inotropes during optimization of the oxygen delivery index (DO_2_I) in high-risk patients submitted to major surgeries are due to fluids, to inotropes, or to the combination of the two is not known. We aimed to investigate the effect of DO_2_I optimization with fluids or with fluids and dobutamine on the 60-day hospital mortality and incidence of complications.

**Methods:**

A randomized and controlled trial was performed in 50 high-risk patients (elderly with coexistent pathologies) undergoing major elective surgery. Therapy consisted of pulmonary artery catheter-guided hemodynamic optimization during the operation and 24 hours postoperatively using either fluids alone (*n *= 25) or fluids and dobutamine (*n *= 25), aiming to achieve supranormal values (DO_2_I > 600 ml/minute/m^2^).

**Results:**

The cardiovascular depression was an important component in the perioperative period in this group of patients. Cardiovascular complications in the postoperative period occurred significantly more frequently in the volume group (13/25, 52%) than in the dobutamine group (4/25, 16%) (relative risk, 3.25; 95% confidence interval, 1.22–8.60; *P *< 0.05). The 60-day mortality rates were 28% in the volume group and 8% in the dobutamine group (relative risk, 3.00; 95% confidence interval, 0.67–13.46; not significant).

**Conclusion:**

In patients with high risk of perioperative death, pulmonary artery catheter-guided hemodynamic optimization using dobutamine determines better outcomes, whereas fluids alone increase the incidence of postoperative complications.

## Introduction

Mortality is unacceptably high in certain groups of surgical patients [1]. Advanced age, extensive surgical trauma, cancer, blood transfusions, and poor nutritional state are conditions reported to be associated with severe changes of the body's defense mechanisms, making the patient highly susceptible to morbidity and mortality [2-4]. Sepsis and multiple organ failure remains the most common cause of death [5].

Some authors have shown that the optimization of the oxygen delivery index (DO_2_I > 600 ml/minute/m^2^), guided by a pulmonary artery catheter, in the perioperative period of high-risk patients determined better survival and less episodes of complications when initiated before the development of organ failure and when therapy produced differences in oxygen delivery [5-11]. The term 'optimization' refers to therapeutic intervention mainly with fluids, inotropic drugs and red blood cells, aiming at a better cardiovascular function anticipating the increase in oxygen demand during surgery. The main objective is to maintain tissue perfusion to minimize the hepatosplanchnic ischemia, thus assuring organic function.

High-risk surgical patients often present a decreased intravascular volume prior to a surgical procedure due to various factors. The importance of fluid replacement in the perioperative period cannot be underestimated [12]. Anesthetic induction still results in increases in systemic vascular capacitance and, to a certain extent, in myocardial depression. Patients with chronic heart failure can face a series of events during prolonged surgery that may end in acute decompensation. Even critically ill patients without preexisting myocardial contractile dysfunction may sustain severe perioperative complications with subsequent acute heart failure [13].

In previous randomized controlled trials in high-risk surgical patients, after the adequate recovery of volemia, either dobutamine or dopexamine have been used with the objective to optimize the cardiac index and/or the oxygen delivery [6-10]. Older patients with existing cardiorespiratory illness undergoing major surgery have a reduced morbidity and mortality when dobutamine is used to maximize oxygen transport [10]. Nevertheless, in certain groups of surgical patients, goal-directed therapy using fluids alone improved the outcome [14-17]. Evidence that optimization of fluid therapy, in the absence of inotropes, reduces mortality in high-risk patients is not available. The primary outcome measure of the present study was to evaluate the effect of both DO_2_I optimization with fluids or with fluids and dobutamine on the 60-day mortality in high-risk general surgery patients. The second outcome measure was the incidence of complications, particularly cardiovascular adverse events.

## Materials and methods

This study, approved by the Institutional Review Board, was carried out in the operating room and the intensive care unit (ICU) (24 beds) of a tertiary hospital. The informed consent to take part in the study was obtained from the patient or from their closest relative. Patients undergoing elective surgeries were admitted to the study if they assigned ≥ 3 points according to a risk scoring system (Table 1) adapted from American College of Cardiology/American Heart Association guidelines [18]. The exclusion criteria were refusal of consent, hemodynamic instability prior to surgery, congestive heart failure, presence of infection, acute myocardial ischemia prior to enrolment, life expectancy lower than 60 days, and disseminated malignancy.

### Measurements of hemodynamic and oxygenation variables

The electrocardiograph, pulse oxymetry and mean arterial pressure (MAP) were monitored continuously during the study period. A pulmonary artery catheter was introduced prior to surgery in the ICU or in the operating room (Balloon Thermodilution Catheter, 7 F, 3 lumen TD, Arrow F; Arrow International, Inc., Reading, PA, USA) and mixed venous blood samples (pulmonary artery) were taken for analysis of the pH, PaO_2_, PaCO_2_, arterial oxygen saturation, mixed venous oxygen  saturation, hematocrit and hemoglobin levels, and lactate (OMNI Modular System AVL Roswell, GA, USA). Cardiac output measurements were obtained using thermodilution methods as previously described [10]. Measurements of the cardiac index, pulmonary artery occlusion pressure (PAOP) and mixed venous and arterial blood gas were obtained directly each hour during the surgery and each 4 hours after admission to the ICU during 24 hours; the other variables were calculated according to standard formulae. The maximum PAOP was defined as the higher value of PAOP obtained during surgery and 24 hours postoperatively.

### Management

The patients were randomized with the use of sealed envelopes (blocks of 10 patients) to either the volume group or the dobutamine group. To induce and maintain anesthesia the following drugs were used: midazolam, 0.05–0.10 mg/kg; etomidate, 0.3 mg/kg; sufentanil, 1 μg/kg (maintenance, 0.01 μg/kg/minute); atracurium, 0.5 mg/kg (maintenance, 0–10 μg/kg/minute); and isoflurane. The therapeutic goals were the same in both groups: maintenance of DO_2_I > 600 ml/minute/m^2^, MAP between 70 and 110 mmHg, PAOP between 12 and 16 mmHg, hematocrit > 30%, arterial oxygen saturation > 94%, and urinary output > 0.5 ml/kg/hour.

**Figure 1:**
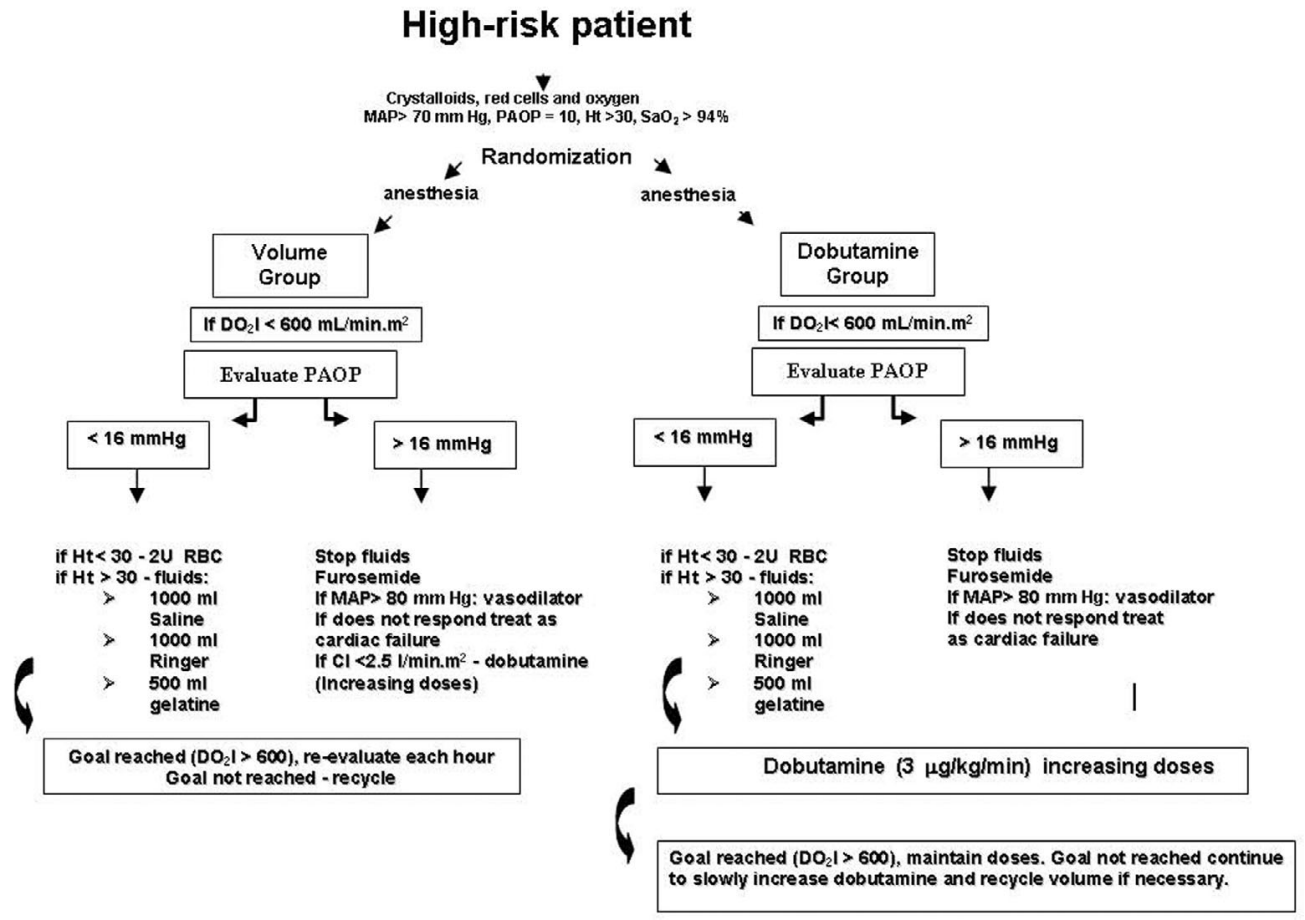
Treatment algorithm. MAP, mean arterial pressure; PAOP, pulmonary artery occlusion pressure; Ht, hematocrit; SaO_2_, arterial oxygen saturation; DO_2_I, oxygen delivery index; RBC, red blood cells; CI, cardiac index.

The patients randomly selected for the volume group were treated with fluids according to the treatment algorithm (Figure 1). The patients randomized to the dobutamine group received the first fluid cycle during 60–90 minutes followed by increasing doses of dobutamine, beginning with 3 μg/kg/minute, until the goal was reached. The patient should receive a new fluid cycle if the PAOP decreased for less than 12 mmHg or if there was a strong clinical suspicion of hypovolemia.

The heart rate, rhythm and MAP were carefully monitored and the dose of dobutamine was decreased or interrupted in the case of hypotension (MAP < 70 mmHg) and/or in the presence of signs of myocardial ischemia (depressed ST segment or inexplicable hypotension or tachycardia). The Acute Physiology and Chronic Health Evaluation II scores were calculated after admission to the ICU [19]. The C-reactive protein serum level (nephelometry) was evaluated after surgery as a marker of inflammation. The dobutamine infusion was maintained for 24 hours in the postoperative period and then was slowly reduced until complete interruption. The mechanical ventilation and weaning were performed according to the ICU routine. Fentanyl and midazolam were used for sedation and analgesia.

### Outcome

The patient was defined as an achiever when DO_2_I > 600 ml/minute/m^2 ^was attained for at least one time point. Patients were followed up for 60 days. Diagnosis of complications was based on predefined criteria. Acute heart failure was designated by the presence of signs of myocardial dysfunction with PAOP > 18 mmHg and cardiac index < 2.2 l/minute/m^2^. Pulmonary edema was considered in the presence of radiological signs of pulmonary edema along with PAOP > 18 mmHg and clinical repercussion leading to prolonged mechanical ventilation/ICU stay or reintubation. Acute myocardial infarction was considered in the presence of electrocardiographic signs of ischemia with an increase of cardiac enzymes and/or segmental changes in the echocardiogram. Arrhythmia was considered when a different cardiac rhythm with hemodynamic repercussions or a need for anti-arrhythmic drugs was recorded. Mesenteric infarction due to acute insufficiency of the splanchnic blood flow was designated by direct visualization during emergent surgery.

Postoperative bleeding was defined as the presence of bleeding requiring new surgical exploration or the transfusion of more than 2 units blood derivatives. Gastrointestinal bleeding was considered as standard. Acute renal failure was defined as an increase greater then two times in the creatinine serum level in the postoperative period in patients with previous normal renal function. Acute respiratory failure was defined as a PaO_2_/FiO_2 _ratio ≤ 200 mmHg and PAOP < 18 mmHg and a need for invasive or non-invasive mechanical ventilation. For nosocomial infections, Centers for Disease Control definitions were used [20]. Postoperative fistulas and dehiscence of anastomosis were determined by visualizing the elimination of intestinal content via drain, wound, or abnormal orifice and by dehiscence of the surgical wound when there is a superficial or deep opening of the wound. The diagnosis of severe sepsis and septic shock were defined according to the American College of Chest Physicians/Society of Critical Care Medicine [21]. An investigator who was unaware of patient allocation by analyzing medical records as well as all radiological and laboratory investigations undertook evaluation of complications retrospectively.

### Statistical analysis

The size of the sample was based on 60-day inhospital mortality rates estimated at 40% for the volume group and 15% for the dobutamine group (assuming that optimization with fluids alone would be the same as no optimization) [6,10]. To have a study power of 80% and a two-sided test with a significance of 0.05, 49 patients would be required in each group. The first statistical evaluation was to be performed when 50% of the patients were enrolled to seek differences either on primary outcomes or on second outcomes. At this point, statistically significant differences were found in major outcomes. It was thought unethical to continue and the study was terminated.

Continuous variables were compared with Student's *t *test. Analysis of variance was used for repeated measurements. When there were significant statistical differences the Bonferroni test was used to detect at which moment the differences occurred. The incidence of complications and mortality rates were evaluated with the relative risk (RR) (95% confidence interval (CI)). *P *< 0.05 was considered statistically significant.

## Results

Over an 18-month period (from May 2002 to July 2004) there were 594 admissions of patients undergoing surgery for postoperative care in the ICU, and 432 of these were elective surgeries. A total of 72 patients (16.5%) were recognized as fulfilling the entrance criteria and 51 patients were enrolled into the study. Twenty-one patients were not enrolled; two because of patient refusal and 19 due to logistic reasons (for example, unavailability of an ICU bed or a theater room, the attending physician's refusal). One patient then had the planned surgical procedure changed to a palliative surgery due to disseminated malignancy and was withdrawn. Fifty patients completed the study: 25 in the volume group and 25 in the dobutamine group.

The demographic data of the patients are presented in Table 2. The therapeutic interventions and perfusion variables are presented in Table 3. In the first 24 hours after ICU admission, the patients in the volume group received significantly more red blood cells than those in dobutamine group (1064 ± 684 ml versus 650 ± 226 ml, respectively; *P *< 0.05). In the volume group, two patients received dobutamine intraoperatively and six patients received dobutamine postoperatively due to a cardiac index lower than 2.5 l/minute/m^2 ^according to the treatment algorithm. Dobutamine was discontinued in five patients in the dobutamine group in the postoperative period either due to tachycardia or arterial hypertension. The percentage of goal-achievers intraoperatively was 28% in the volume group (7/25) in comparison with 84% in the dobutamine group (21/25) (RR = 0.33, 95% CI = 0.17–0.63). In the ICU, significantly less patients in the volume group (16/25, 64%) than in the dobutamine group (22/25, 88%) were goal-achievers (RR = 0.73; 95% CI = 0.52–1.00). At the end of the optimization therapy there were 76% achievers in the volume group (19/25) and 96% in the dobutamine group (24/25) (RR = 0.79, 95% CI = 0.62–1.00).

**Figure 2:**
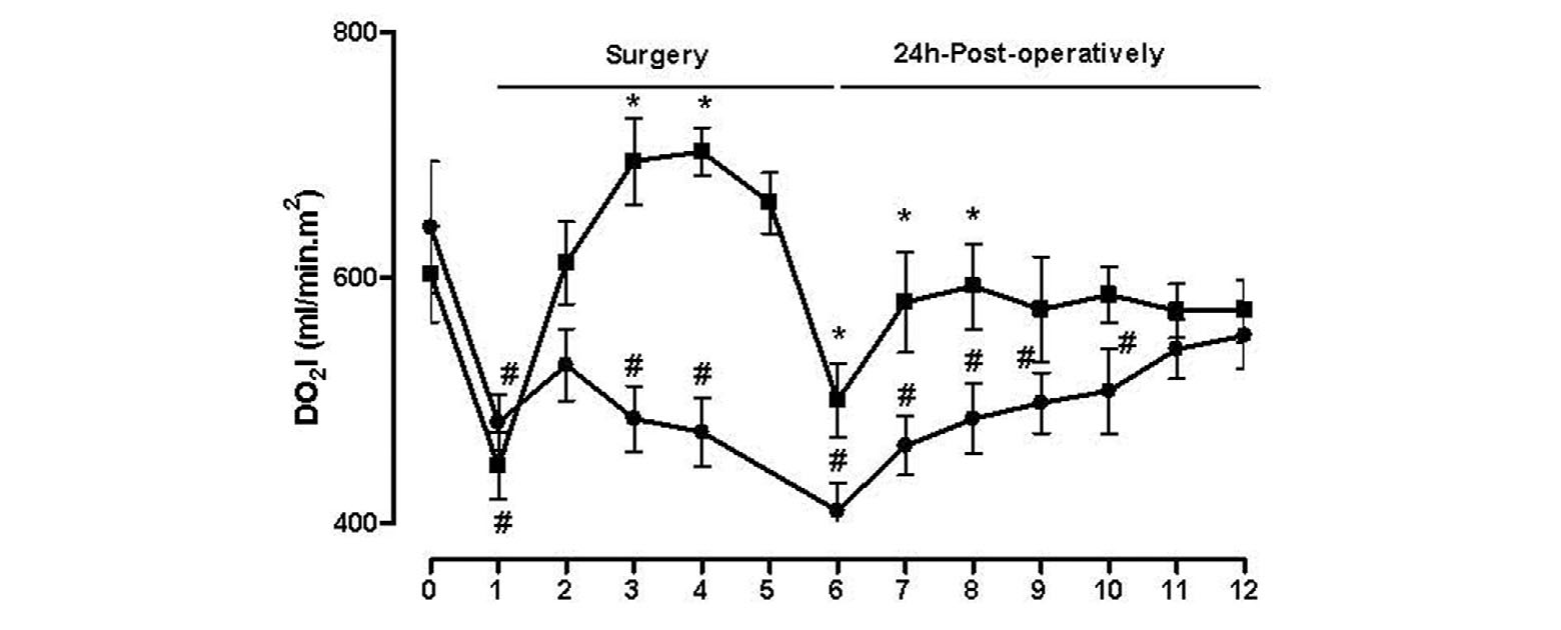
Oxygen delivery index during surgery and postoperatively. Oxygen delivery index (DO_2_I) during surgery and postoperatively for the volume group (●) and the dobutamine group (■). *P < 0.05 versus volume group, ^#^*P *< 0.05 versus baseline. Results presented as the mean and standard error or the mean. 0, preoperative; 1, 30 min intraoperatively; 2, 2 hours intraoperatively; 3, 4 hours intraoperatively; 4, 6 hours intraoperatively; 5, 8 hours intraoperatively; 6, 0 hours postoperatively; 7, 4 hours postoperatively; 8, 8 hours postoperatively; 10, 12 hours postoperatively; 11, 16 hours postoperatively; 12, 24 hours postoperatively.

Figure 2 shows the temporal pattern of the DO_2_I during surgery and postoperatively for the volume and dobutamine groups. An important DO_2_I reduction was seen after the start of anesthesia in both groups. While a recovery was seen in the dobutamine group, however, the DO_2_I remained significantly lower in the volume group in comparison with baseline, with a statistically significance difference at 4 and 6 hours intraoperatively and at 0, 4, 8, 12 and 16 hours postoperatively (*P *< 0.05 for all). The dobutamine group had a significantly higher DO_2_I than the volume group during surgery (at 4 hours, 695 ± 176 versus 485 ± 134 ml/minute/m^2^; at 6 hours, 703 ± 99 versus 474 ± 134 ml/minute/m^2^; dobutamine group versus volume group, *P *< 0.05 for both) and postoperatively (at 0 hours, 500 ± 151 versus 410 ± 113 ml/minute/m^2^; at 4 hours, 580 ± 204 versus 463 ± 122 ml/minute/m^2^; at 8 hours, 593 ± 172 versus 485 ± 144 ml/minute/m^2^; dobutamine group versus volume group, *P *< 0.05 for all).

The temporal patterns of the cardiac index, left ventricular stroke work index (LVSWI) and PAOP during surgery and postoperatively are presented in Table 4. The LVSWI significantly decreased in both groups during the operation and was significantly lower in the volume group (41 ± 13 g/m/m^2^) than in the dobutamine group (48 ± 9 g/m/m^2^) at 4 hours. The levels of PAOP were significantly higher in the volume group at 6 hours postoperatively in comparison with the dobutamine group (11 ± 3.3 versus 8 ± 1.6 mmHg, *P *< 0.05). The maximum PAOP was significantly higher in the volume group than in the dobutamine group, both intraoperatively and postoperatively (14.4 ± 3.8 versus 12.4 ± 2.9 mmHg and 16.0 ± 3.1 versus 14.1 ± 3.4 mmHg, respectively; *P *< 0.05).

### Postoperative complications

Cardiovascular complications in the postoperative period occurred significantly more frequently in the volume group (13/25, 52%) than in the dobutamine group (4/25, 16%) (RR = 3.25, 95% CI = 1.22–8.60) (Table 5). The prevalence of infection was similar in both groups (volume group, 28%; dobutamine group, 48%; not significant). Complications occurred in 74% of the achievers in the volume group (14/19) and in 58% of the achievers in the dobutamine group (14/24) (not significant).

### Mortality

There were no significant differences in 28-day or 60-day mortality. The 28-day mortality rates were 20% in the volume group and 8% in the dobutamine group. The 60-day mortality rates were 28% in volume group and 8% in dobutamine group (RR = 3.0, 95% CI = 0.67–13.46).

## Discussion

Fluids improve morbidity and mortality when combined with inotropes during major surgeries. Randomized controlled trials evaluated the effect of perioperative optimization on mortality in high-risk surgical patients using either dobutamine or dopexamine to improve the DO_2_I [6-10]. These studies did not investigate the effects of fluids alone. In the present study there was a significant reduction in postoperative complications, particularly cardiovascular complications, and a nonsignificant reduction in mortality in the group optimized with dobutamine and fluids in comparison with the group optimized with fluids alone. Nevertheless, significantly more patients in the dobutamine group were goal-achievers, suggesting an important role of the inotrope in the optimization therapy in this group of high-risk patients submitted to elective surgeries.

Unexpectedly, despite the use of two different treatment algorithms driven to obtain a DO_2_I > 600 ml/minute/m^2^, both groups had received similar amounts of fluids at the end of the optimization therapy. The presence of decreasing values of the LVSWI suggests contractility problems in both groups. A better recovery of the LVSWI was seen in the dobutamine group during the operative trauma. By counteracting the adverse events of the fluids, dobutamine probably made a more generous fluid infusion possible alongside fewer complications in this group. In contrast, significantly higher values of the maximum PAOP in response to fluid challenges suggested that poor cardiovascular reserves limited the fluid infusion in the volume group.

The mechanism of the protective effect of dobutamine is still not completely elucidated. Impaired tissue perfusion due to hypovolemia, disturbed vasoregulation, and myocardial dysfunction contributes to multiple organ dysfunctions that can be prevented by the prompt compensation of the oxygen debt by maintaining supranormal values intraoperatively and in the very early postoperative period [6,10,22,23]. Significantly, more patients were achievers in the dobutamine group than in the volume group, especially in the intraoperative period. If therapy-induced differences in oxygen delivery determine better outcomes, then our results suggest that the use of inotropes is necessary during the optimization therapy in this group of high-risk patients [11]. It is also possible that some beneficial effects observed were related to inotropes' therapy effects in regulating inflammation. Higher levels of C-reactive protein, an inflammation marker, have been detected in volume group; however, the study was precociously terminated and the results were not statistically significant [24]. Nevertheless, dobutamine improves microcirculatory perfusion and increases splanchnic blood flow, which could prevent more inflammation secondary to the tissue hypoxia and to the translocation of bacterial products or endotoxin [25,26].

The hospital stay was slightly longer in the dobutamine group despite the fact that the complications and mortality rates were lower in this group. Other authors reported a significant reduction in hospital stay mainly due to the reduction of postoperative infection with postoperative goal-directed therapy using dopexamine to attain a DO_2_I > 600 ml/minute/m^2 ^– a finding different from the present study [23]. The choice of inotrope may play a role. Dopexamine may confer an additional advantage to fluid optimization by reducing the effect of infective complications. In different studies on optimization, both inotropes (dobutamine and dopexamine) produced the desired preoperative increase in oxygen delivery. Only dopexamine, however, seems to reduce the hospital stay and infectious complications.

Several potential limitations in our study require comment. First, blinding was not possible for evaluating outcome and there was a small imbalance in comorbidities that could determine case-mix differences between groups.

Second, the volume group received about two times more packed red blood cells than the dobutamine group. It is probable that this group needed more transfusions due to either the presence of defective tissue perfusion signs or to differences in blood losses between groups [27]. Transfusion of blood derivatives is an independent risk factor for worse outcome [28]. The complications related to the transfusion of blood derivatives are infectious most of the time, however, which in fact did not occur in the present study.

Third, it is possible that the therapeutic approach in the volume group was not aggressive enough once many patients failed to achieve the targeted DO_2_I levels. Indeed, the PAOP was between 8 and 13 mmHg, although the algorithm suggested that it had to be pushed up to 16 mmHg. The significantly higher values of maximum PAOP seen in this volume group, however, suggest the presence of a poor cardiovascular reserve limiting fluid challenge more than the presence of hypovolemia.

Finally, it is possible that the sample size calculation of 40% mortality rate for this population of elective high-risk surgery patients was overestimated and the study was underpowered. We believe, however, that the association of a median of four risk surgery criteria determines a higher risk population than those reported by other authors [7-9,29].

Fluid optimization alone reduced complications and improved recovery times in certain groups of surgical patients [14-16]. Associations between postoperative fluid overload and poor survival have been shown in other studies, however, and better outcomes after restrictive fluid resuscitation regimens after surgery were reported [30-32]. The numbers of cardiopulmonary and tissue-healing complications were significantly reduced when a restricted fluid regimen in the perioperative period was used in a randomized multicenter trial after colorectal surgery [30]. Another prospective study comparing the use of restrictive versus liberal fluid management on postoperative outcome in patients undergoing elective intraabdominal surgery showed less complications in the restrictive group than in liberal group [31]. We must consider, however, that these studies have been carried out in groups of less severely ill patients and with better cardiovascular reserve. Furthermore, different therapeutic regimens and patient populations make the comparison of these studies difficult.

The cardiovascular depression was an important component of the hemodynamic response in the perioperative period in this group of patients. The reductions observed in the DO_2_I and in the myocardial contractility evaluated by the LVSWI were dramatic, especially at two important points of the perioperative period: after the induction of anesthesia and after transport to the ICU. These findings suggest that special attention should be directed to the hemodynamic support at these moments. In addition, patient safety may be enhanced by increasing the use of beta-blockers in high-risk patients and perioperative treatment with beta-blockers is now widely advocated [33]. Up to now, the hemodynamic effect of beta-blocking agents on dobutamine infusion has been controversial [34,35]. In addition, the best fluid replacement therapy, either restrictive or liberal in association with an inotrope, must be the subject of future studies.

## Conclusion

Pulmonary artery catheter-guided hemodynamic optimization using dobutamine determines better outcomes, whereas fluids alone increase the incidence of postoperative complications in patients with high risk of perioperative death.

## Key messages

• 	Preventing perioperative tissue oxygen debt contributes to a better outcome in high-risk surgical patients.

• 	The cardiovascular depression was an important component of the hemodynamic response in the perioperative period in this group of patients.

• 	In major elective surgery, pulmonary artery catheter-guided hemodynamic optimization using dobutamine and fluids determines better outcomes, whereas fluids alone increase the incidence of postoperative complications in patients with a high risk of perioperative death.

## Abbreviations

CI = confidence interval; DO_2_I = oxygen delivery index; FiO_2_ = fraction of inspired oxygen; ICU = intensive care unit; LVSWI = left ventricular stroke work index; MAP = mean arterial pressure; PaCO_2_ = partial pressure of carbon dioxide; PaO_2_ = partial pressure of oxygen; PAOP = pulmonary artery occlusion pressure; RR = relative risk.

## Competing interests

The authors declare that they have no competing interests. All authors take full responsibility for the integrity of the data and accuracy of the analysis.

## Authors' contributions

SML, FRL and CAP were responsible for the study design, data analysis and manuscript drafting. FRL, DSP, AEY, PAA and EFS were responsible for anesthesia and administering the protocol during surgery. NEO, PS, MAS, ACC Jr, MMQ and SPT were responsible for administering the protocol in the ICU. HSS was responsible for analysis of postoperative complications. GSC, DSP, AEY, PAA and HSS were responsible for patient recruitment.

## Figures and Tables

**Table 1:** Risk scoring system (adapted from American College of Cardiology/American Heart Association guidelines)

Score	Risk factor
High-risk surgery (1 point)	Gastrectomy Pancreatectomy Total colectomy Total esophagectomy Another long surgical procedure associated with large fluid shifts or blood losses
Minor clinical predictor (1 point)	Diabetes (defined as standard taking medication or not) Abnormal electrocardiogram (left ventricular hypertrophy, left bundle-branch block, ST-T abnormalities, atrial fibrillation) Low functional capacity (inability to climb one flight of stairs with a bag of groceries) Arrhythmia (using drugs) History of stroke Arterial hypertension (Difficult controlling).
Intermediate clinical predictor (2 points)	Age (>60 years) Angina (class I or II, Canadian Cardiovascular Society Classification) or previous infarctus or Q waves Compensated or previous decompensated heart failure (defined as standard) Chronic hepatic failure (defined as standard) Chronic renal failure (preoperative creatinine > 2.0 mg/dl or need for dialysis) Chronic obstructive pulmonary disease (defined as standard) or severe respiratory illness resulting in functional limitation
Major clinical predictor (3 points)	Angina (class III or IV, Canadian Cardiovascular Society Classification) Severe arrhythmias (advanced heart block, ventricular arrhythmia in the presence of cardiopathy or supraventricular arrhythmia with tachycardia) Severe valvopathy (severe valvular regurgitation with reduced left ventricular function)

**Table 2:** Baseline characteristics of patients in the volume group and the dobutamine group

	Volume group	Dobutamine group
Number of patients	25	25
Male (%)	69	76
Age	69.9 ± 8.4	67.6 ± 7.5
Acute Physiology and Chronic Health Evaluation II score	14.4 ± 6.2	12.4 ± 6.5
Risk Scoring System (points)	4.25 ± 1.22	4.44 ± 1.53
Aged > 60 years	23 (92%)	20 (80%)
Cancer	24 (96%)	25 (100%)
Arterial hypertension	16 (64%)	13 (52%)
Coronary artery disease	6 (24%)	5 (20%)
Diabetes mellitus	5 (20%)	2 (8%)
Chronic obstructive pulmonary disease	4 (16%)	0 (0%)
Arrhythmia	3 (12%)	2 (8%)
Cerebral vascular accident	1 (4%)	0 (0%)
Compensated heart failure	2 (8%)	3 (12%)
Chronic renal failure	0 (0%)	1 (4%)
Type of surgery		
Colorectal	9	5
Hepatobiliary	1	1
Duodenopancreatectomy	2	5
Esofagectomy	1	3
Gastrectomy	6	5
Urology	5	4
Other	1	2

**Table 3:** Therapeutic interventions and changes in perfusion variables during optimization

	Volume group	Dobutamine group
Intraoperatively		
Operation time (min)	315 ± 109	331 ± 132
Total volume (l/hour)	1.31 ± 0.54 (0.97/1.21/1.54)	1.35 ± 0.43 (1.05/1.29/1.65)
Red blood cells (ml)	609 ± 244 (480/600/600)	713 ± 458 (500/600/650)
Number of transfused patients (%)	11 (44%)	12 (48%)
Dobutamine doses (μg/kg/minute)	3.5 ± 0.7	5.5 ± 1.8
Dobutamine (number of patients)	2 (8%)	25 (100%)*
Initial serum lactate (mEq/l)	1.3 ± 0.6	1.3 ± 0.5
Final serum lactate (mEq/l)	1.7 ± 0.7	1.9 ± 0.9
Initial mixed venous oxygen saturation	83 ± 4	81 ± 6
Final mixed venous oxygen saturation	86 ± 4	86 ± 7
Number of goal-achievers	7 (28%)	21 (84%)*
Intensive care unit		
Total volume (l/hours)	0.29 ± 0.12	0.28 ± 0.18
Red blood cells (ml)	1064 ± 684 (450/600/900)	650 ± 226* (600/600/600)
Number of transfused patients (%)	7 (28%)	6 (24%)
Dobutamine doses (μg/kg/minute)	5.7 ± 2.3	6.1 ± 3.4
Dobutamine (number of patients)	6 (24%)	20 (80%)*
Sodium nitroprusside	6 (24%)	2 (8%)
Initial serum lactate (mEq/l)	2.5 ± 1.6	2.2 ± 0.8
Final serum lactate (mEq/l)	1.9 ± 2.0	1.3 ± 0.4
Initial mixed venous oxygen saturation	76 ± 13	78 ± 9
Final mixed venous oxygen saturation	78 ± 9	77 ± 8
Number of goal-achievers	16 (64%)	22 (88%)*

Data presented as the absolute value (%) or as the mean ± standard deviation. *P < 0.05 versus volume group.

**Table 4:** Hemodynamic and perfusion variables during surgery and postoperatively

	0	1	2	3	4	5	6	7	8	9	10	11	12
Cardiac index (l/minute/m^2^)													
Volume group	3.6/1.1 (25)	3.2/1.1 (25)	3.3/0.8 (22)	3.5/0.6 (15)	3.4/0.8 (13)	3.9/0.8 (4)	2.5/0.7	2.9/0.8	3.1/0.9	3.2/0.8	3.5/0.9	3.8/1.2	3.7/0.6
Dobutamine group	3.4/0.7 (25)	3.0/0.8 (25)	4.0/1.0^*# ^(22)	4.6/1.2* ^# ^(13)	4.5/0.5* ^# ^(10)	4.6/0.7^# ^(8)	3.0/0.9*	3.6/1.0*	3.8/1.0*	3.9/0.8* ^#^	3.9/0.7^#^	4.0/0.8^#^	4.0/0.9^#^
Left ventricular stroke work index (g/m/m^2^)													
Volume group	65/22 (25)	43/12^# ^(25)	43/12^# ^(22)	41/13^# ^(15)	46/12^# ^(10)	45/11 (4)	37/13^#^	37/15^#^	41/23^#^	42/28^#^	46/18^#^	45/22^#^	42/18^#^
Dobutamine group	58/15 (25)	43/13^# ^(25)	49/17 (22)	48/9* ^# ^(13)	50/14 (10)	49/18 (8)	39/18^#^	43/20^#^	41/19^#^	42/21^#^	41/20^#^	43/17^#^	42/16^#^
Pulmonary artery occlusion pressure (mmHg)													
Volume group	12/4.3 (25)	12/4.3 (25)	10/3.0 (22)	10/3.0 (14)	11/3.3 (12)	8/1.1 (3)	9/4.1	11/4.7	13/4.5	11/4.6	12/3.5	12/3.3	12/3.9
Dobutamine group	11/4.0 (25)	12/3.3 (25)	10/2.9 (22)	9/2.5 (13)	8/1.6* ^# ^(10)	10/1.1 (8)	9/3.3	10/3.9	11/3.5	11/4.4	11/4.4	11/3.7	12/3.3
Lactate (mEq/l)													
Volume group	1.3/0.6	1.3/0.6	1.2/0.5	1.7/0.6	2.5/1.6	2.8/2.0	2.5/1.6	2.8/1.9	2.0/1.9	2.2/2.0	2.1/2.4	1.9/1.8	1.9/2.0
Dobutamine group	1.3/0.5	1.4/0.8	1.9/0.6	1.2/0.4	2.2/0.8	1.9/0.8	2.2/0.8	1.9/0.8	1.6/0.6	1.6/0.8	1.7/0.8	1.3/0.8	1.3/0.4
Mixed venous oxygen saturation (%)													
Volume group	83/4	-	86/3	86/5	86/5	86/2	76/13	73/11	76/15	77/10	77/10	77/11	78/9
Dobutamine group	81/6	-	86/2	87/5	85/6	83/11	78/9	80/10	80/9	77/11	79/7	77/8	77/8

Results presented as mean/standard deviation. 0, preoperative; 1, 30 minutes intraoperatively; 2, 2 hours intraoperatively; 3, 4 hours intraoperatively; 4, 6 hours intraoperatively; 5, 8 hours intraoperatively; 6, 0 hours postoperatively; 7, 4 hours postoperatively; 8, 8 hours postoperatively; 10, 12 hours postoperatively; 11, 16 hours postoperatively; 12, 24 hours postoperatively. *P < 0.05 versus volume group, ^#^*P *< 0.05 versus baseline.

**Table 5:** Outcome

	Volume group	Dobutamine group
Cardiovascular complications		
Acute heart failure	2	0
Pulmonary edema	8	3
Acute myocardial infarction	0	1
Cardiac arrhythmia	2	0
Mesenteric infarction	1	0
Number of patients with cardiovascular complications (%)	13 (52%)	4 (16%)*
Infectious complications		
Pneumonia	5	7
Peritonitis	3	3
Urinary tract infection	1	2
Fistula	1	2
Severe sepsis/septic shock	7	4
Number of patients with infectious complications (%)	7 (28%)	12 (48%)
Other complications		
Postoperative bleeding	1	0
Upper gastrointestinal bleeding	0	1
Acute renal failure	3	1
Acute respiratory failure	5	2
Number of patients with other complications (%)	9 (36%)	4 (16%)
Total number of complications	39	26
Number of patients with complications (%)	17(68%)	14(56%)
Number of achievers with complications (%)	14 (74%)	14 (58%)
C-reactive protein on day 1 (mg/dl)	18 (11-23)	13 (10-18)
Median duration of intensive care unit stay (days)	3 (2.0-6.0)	3(3.0-8.5)
Median duration of hospital stay (days)	14 (8.5-8.5)	20 (14.5-32)
Mortality rate		
28 days	5 (20%)	2 (8%)
60 days	7 (28%)	2 (8%)

Data presented as the absolute value (%) or as the median (interquartile range). *P < 0.05 versus volume group.
